# TIPE2 Inhibits MGD Inflammation by Regulating Macrophage Polarization

**DOI:** 10.3390/jpm13030492

**Published:** 2023-03-09

**Authors:** Songjiao Zhao, Yankun Shen, Shinan Wu, Yi Shao, Ruize Shi, Yan Yan, Hui Zhao

**Affiliations:** 1 Department of Ophthalmology, Shanghai General Hospital, Shanghai Jiao Tong University School of Medicine, Shanghai 200080, China; 2 National Clinical Research Center for Eye Diseases, Shanghai 200080, China; 3 Shanghai Key Laboratory of Ocular Fundus Diseases, Shanghai 200080, China; 4 Shanghai Engineering Center for Visual Science and Photomedicine, Shanghai 200080, China; 5 Shanghai Engineering Center for Precise Diagnosis and Treatment of Eye Diseases, Shanghai 200080, China; 6 Department of Ophthalmology, Jiangxi Branch of National Clinical Research Center for Ocular Disease, The First Affiliated Hospital of Nanchang University, Nanchang 330006, China

**Keywords:** meibomian gland dysfunction, macrophage, TIPE2, polarization

## Abstract

Background: The aim of this study was to decide the role of the polarization of macrophages regulated by tumor necrosis factor-α (TNF-α)-induced protein 8-like 2 (TIPE2) in meibomian gland dysfunction (MGD). Methods: Firstly, the secretory function of the meibomian gland (MG) in apolipoprotein E knockout (ApoE^-/-^) MGD mice and normal mice was detected by oil red staining. Then, the expression levels of markers of M1 and M2 macrophages were detected by immunofluorescence staining in MGD, normal mice, and mild and severe MGD corpses to decide the role of M1 and M2 macrophages in MGD inflammation. Meanwhile, the expression levels of TIPE2 in MGD mice and MGD patients were detected by immunofluorescence staining, and the correlations among TIPE2, M1 and M2 macrophages were analyzed by immunofluorescence double staining in MGD mice and MGD patients. Furthermore, lipopolysaccharide (LPS) and interleulkin-4 (IL-4) were used to induce M1 and M2 polarization of macrophages, and the mRNA level of TIPE2 was detected in M1 and M2 macrophages. Results: Oil red staining showed that eyelid fat congestion was more severe in (ApoE^-/-^) MGD mice than in normal mice, and the M1 macrophage was the primary inflammatory cell infiltrated in (ApoE^-/-^) MGD mice (*p* < 0.05). The results of the immunofluorescence staining showed that the infiltration of macrophages in MGD mice was more obvious than that in the normal group, and M1 macrophage was the dominant group (*p* < 0.05). Similar to the results of the MGD mouse model, more macrophage infiltration was observed in MGD patients’ MG tissues, and there were more M1 cells in the severe group than in the mild group (*p* < 0.05). Moreover, the expression of TIPE2 was positively correlated with the expression of M2 macrophages in MGD patients and mice MG tissues (*p* < 0.05). The expression of TIPE2 mRNA in LPS-induced M1 macrophages declined, while the expression of TIPE2 mRNA in IL-4-induced M2 macrophages increased (*p* < 0.05). Conclusion: M1 macrophage was the dominant group infiltrated in the MG tissue of MGD, and TIPE2 is a potential anti-inflammatory target for preventing the development of MGD by promoting the M2 polarization of macrophages.

## 1. Introduction

**Figure 1:**
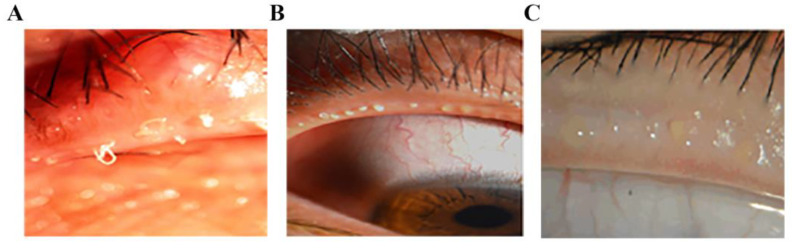
Typical clinical images of MGD patients. The photos of severe MGD (**A**,**B**) and mild MGD (**C**) patients show that the meibomian gland opening was blocked by white keratin and protruded, and the extruded secretion was like foam, granule or toothpaste.

Meibomian gland dysfunction (MGD) is a chronic, non-specific inflammation of the meibomian glands, characterized by the obstruction of meibomian ducts or the abnormal secretion of meibomian glands. In MGD, glandular lipid secretion is damaged and the ocular surface cannot maintain the stability of tear film. Rapid evaporation of tear film and increased osmotic pressure of tear film are the main causes of hyper-evaporative dry eye (Figure 1) [1].

MGD causes changes in tear film composition and affects ocular surface health, which is the main cause of evaporative dry eye. The main manifestations are increased tear evaporation, gradual hyperosmolar, inflammation, damage to ocular surface epithelial cells [2], severe damage to the cornea, and even blindness. The survey portrayed that the incidence of MGD in the Asian population is very high (49.2−69.3%), and is one of the most common forms of eye disease in Asia [3]. MGD is currently incurable and requires chronic disease treatment. The cost of MGD treatment in the United States and Japan has reached USD 55 billion per year [4]. The control of inflammation plays a crucial role in the treatment of MGD.

Environmental pollution, smoking, blepharitis, lack of sleep and other factors can lead to MGD [5]. Its main pathological changes are excessive keratinization of the glandular ducts and an increased viscosity of eyelid fat, which cause blockage of the MG opening, the abnormal differentiation of MG epithelial cells, increased inflammatory mediators, and the infiltration of neutrophils, which in turn cause the blockage of MG glandular ducts [6]. Studies have shown that the occurrence and development of MGD is accompanied by the infiltration of macrophages [7]. Macrophages belong to leukocytes, which are phagocytic cells specialized by the innate immune system. Together with neutrophils, they become the first responders to infection, participating in the recognition, phagocytosis and degradation of cell debris and pathogens, maintaining tissue homeostasis and tissue repair and remodeling [8]. Previous studies on the pathogenesis of MGD have mainly focused on the response of MG to inflammatory stimuli, the anti-inflammatory effect of meiborate, the “flora imbalance” of the eyelid margin, and the infiltration of central granulocytes. Although macrophages have been proven to infiltrate MGD tissues, no relative study has been reported about the specific function and mechanism of different activated macrophages in the immune response of MGD progression [9,10,11].

TIPE2 is a cytoplasmic protein that exists in myeloid, lymphoid and liver cells, and is expressed differently at different stages of cell development. With the deepening of research, TIPE2 is found to be a reversed coordinator of immunity that is involved in the stable immune state, expressed in both immune and non-immune tissues, and is an ideal biomarker [12]. In the study of ophthalmic diseases, TIPE2 acts as an essential character in the inflammation of choroid. Suo et al. [13] observed the expression of TIPE2 in the cytoplasm and nucleus of retinal pigment epithelial cells under normal and inflammatory conditions. With the development of inflammatory response, the cell activity decreased and the expression of TIPE2 decreased. It is clear that the immune function of macrophages is concerned with the standard of TIPE2. After LPS was used to entice mouse bone-marrow-derived macrophages to M1 macrophages, the expression level of TIPE2 was observed to decrease. After IL-4 was induced to M2 macrophages, the standard of TIPE2 was observed to increase [14]. However, the role of TIPE2 in the polarization of macrophages and the immune response of MGD progression is still unknown. In this study, an ApoE^-/-^ MGD mouse model and MGD cadaveric MG tissue were used to explore the characters of macrophages (M1/M2) and the distribution of TIPE2 in the progression of MGD, verifying the regulation of TIPE2 in macrophage polarization around MG in the immune response in order to provide new anti-inflammatory targets for MGD treatment.

## 2. Materials and Methods

Animal model

In this study, an MGD model of ApoE^-/-^ mice was used which showed MGD symptoms at 5 months of age. Based on this, a sampling time point for the MGD model of ApoE^-/-^ mice was developed. Normal mice of the same age were used as a control group. MG tissues and secretions were collected from the ApoE^-/-^ MGD mouse model (1, 3, 5, and 7 months old). MG tissues in each group were divided into three parts, which were prepared into frozen sections and mRNA was extracted for detection. All studies were conducted in line with the declaration of the Association for Visual and Ophthalmic Research on the use of animals in ophthalmology and optical experiments, and were approved by the Animal Ethics Committee of Shanghai Jiao Tong University.

MGD corpse model

None of the 4 MGD cadaveric donors had known systemic diseases. Four donors were divided into two groups: two with mild MGD and two with severe MGD. The donor’s medical history and eye history were identified and collected before the tarsus was removed from the upper eyelid of the fresh corpse. MG tissues and secretions from each group of donors were collected. The MG tissues of each group were divided into three parts and prepared into frozen sections for detection. The removal of donor cornea and tarsal plate was performed by ophthalmologists according to the National Industry Standards for Ophthalmic Bank Management in China. The use of human tissue in the study complied with the provisions of the Helsinki Declaration.

Histology

The meibomian gland tissues were collected and embedded in optimal cutting temperature (OCT) compound or paraffin. Then, the tissues were cut into sagittal sections (5 μm thick) and stored at −80 °C (frozen sections) or room temperature (paraffin sections).

Oil red staining

Mice frozen MG portions were fastened in 4% paraformaldehyde for 10 min, rinsed in phosphate-buffered saline (PBS) for 5 min, and dyed in prepared oil red O solution for 10 min. After washing with PBS for 5 min, the portions were counterstained with hematoxylin and installed in 90% glycerol. The MG eyelid fat and inflammatory cell infiltration of the two groups of mice were observed to determine whether the modeling was successful.

Immunofluorescence staining

Mice inflammatory cells, M1 macrophage markers (CD45, F4/80, CD86, CD68) and M2 macrophage markers (CD163), and human M1 macrophage markers (CD45, F4/80) and M2 macrophage markers (ARG1, YM1) were detected using an immunofluorescence technique. Thus, the dominant macrophage type in this process could be determined. Frozen sections of MGD mice and human model MG tissues were scanned using a PANNORAMIC (3DHISTECH, Hungary) panoramic section scanner and observed with CaseViewer software (2.4 version, 3DHISTECH, Hungary). Halo analysis software (v3.0.311.314, Indica labs, USA) was utilized to quantify the amount and area of positive cells in the target area of MGD mice slices. Thus, the expression changes in TIPE2 in different types of macrophages in MGD meibomian glands were qualitatively and quantitatively observed.

Cell culture and passage of RAW246.7 cell line

RAW 246.7 cell line is a mature immortalized mouse macrophage cell line. The culture system used DMEM medium, 10% fetal bovine serum and 1% double antibody, and the liquid was changed every two days. When the cells grew to 70–80%, they were passaged at 1:3, and P3 cells were used for subsequent experiments.

Transformation of RAW 246.7 cells induced by IL-4 and LPS

IL-4 and LPS were used in four concentration gradients of 0 ng/mL (control), 10 ng/mL, 20 ng/mL and 40 ng/mL, and four time gradients of 6 h, 12 h, 24 h and 48 h were chosen, with three replicate samples in each group.

TIPE2 mRNA extraction, concentration determination and reverse transcription

Then, 200 µL chloroform was added to a 1.5 mL Eppendorf tube, mixed upside-down, left indoors for 2 min (min), and then centrifuged at 14,000 r/min at 4 °C for 15 min. Then, 500 µL of the upper water phase was absorbed into the new Eppendorf tube, and 600 µL of isopropyl alcohol was added, mixed, and placed at −80 °C for alcohol precipitation for 15 min. The samples were centrifuged at 4 °C and 12,000 r/min for 10 min, and the supernatant was discarded with the pipette. Then, 500 uL 75% ethanol with diethypyrocarbonate (DEPC) was added to each tube, washed fully with vortex, and centrifuged at 12,000 r/min at 4 °C for 5 min. After centrifugation at 12,000 r/min for 5 min, the supernatant was abandoned and the residual liquid was dried by instantaneous centrifugation. Subsequently, it was placed in a fume hood for drying for 10 min until the precipitate became transparent. The precipitate was dissolved in 20 µL DEPC and reserved in a refrigerator at −80 °C for subsequent steps. The extracted mRNA concentration was determined using a spectrophotometer and diluted to a range of 400–600 ng/mL with DEPC. Reverse transcription was undertaken according to the 20 µL system. Firstly, the required premix solution was calculated according to the number of samples, and then the sample was added. Finally, the temperature settings of 25 °C 10 min, 42 °C 60 min, and 70 °C 10 min were used for reverse transcription. The transcribed samples were stored at −20 °C.

RNA extraction and Quantitative real-time PCR

MG tissues were isolated with skin, muscle and removed conjunctiva. The total RNA of the MG tissue was extracted using TRIzol reagent (Invitrogen, Carlsbad, CA, USA), and genomic DNA was removed using DNaseI (TaKara, Shiga, Japan). An ND-2000 spectrophotometer (NanoDrop Technologies, Seattle, WA, USA) was used for RNA quantification. The RNA samples with an eligible value of OD260/280 (1.8–2.2) and OD260/230 (≥2.0) were used for further experimentation. qRT PCR was conducted according to a 10 uL system. First, the premix was configured, and three duplicate samples were added to each sample. Each sample measured ACTIN and TIPE2 genes, and was finally placed in the electrolyte analyzer (Roche, Basel, Switzerland) for qRT-PCR. The thermal analysis conditions used were 95 °C 10 min, 95 °C denaturation 10 s, 60 °C annealing extension 30 s, and 40 cycles. Finally, the data was analyzed using Roche software.

Data analysis

Student’s unpaired *t*-test was conducted on the density of inflammatory cells around MG in normal mice and MGD mice using GraphPad Prism software (San Diego, CA, USA). Student’s unpaired *t*-test was performed on the expression of TIPE2 mRNA in M1 and M2 macrophages around MG tissues of IL-4- and LPS-induced MGD mice. A *p*-value less than 0.05 signified the difference was of statistical significance.

## 3. Results

### 3.1. The Role of Macrophages in MGD

**Figure 2:**
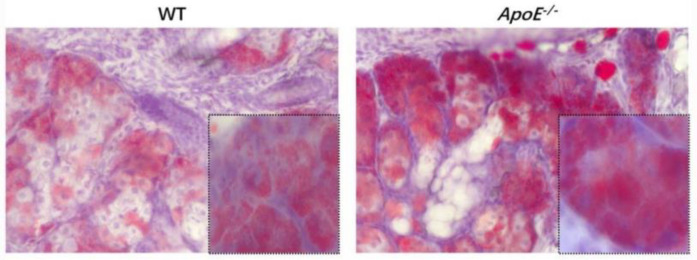
Oil red staining results of normal and ApoE^-/-^ MGD mice. The results showed that ApoE^-/-^ MGD mice had more obvious congestion of eyelid fat and a greater infiltration of inflammatory cells than WT mice.

**Figure 3:**
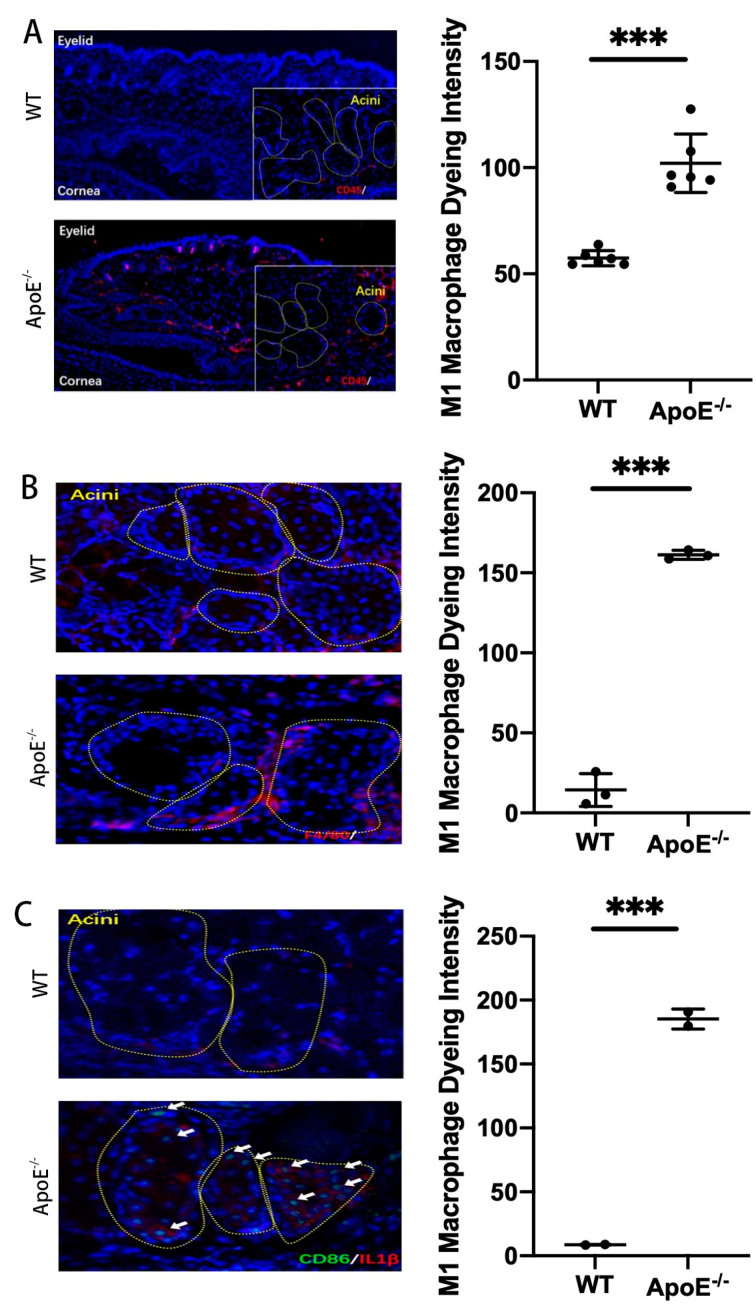
Immunofluorescence staining of macrophage markers in MGD mice. The acini of meibomian gland were signed by dashed boxes. (**A**) The results showed that the expression levels of CD45 (marker of M1 type) were significantly increased in ApoE^-/-^ mice (MGD mice) than those in WT mice (*p* < 0.0001). (**B**) The results showed that the expression levels of F4/80 (marker of M1 type) were significantly increased in ApoE^-/-^ mice (MGD mice) compared to those in WT mice (*p* < 0.0001). (**C**) The results showed that the expression levels of CD86 (marker of M1 type, signed by white arrows) were significantly increased in ApoE^-/-^ mice (MGD mice) compared to those in WT mice (*p* = 0.0010). No significant difference was found in the expression of IL1β (marker of M1 type) between WT and ApoE^-/-^ mice (*** *p* < 0.001).

**Figure 4:**
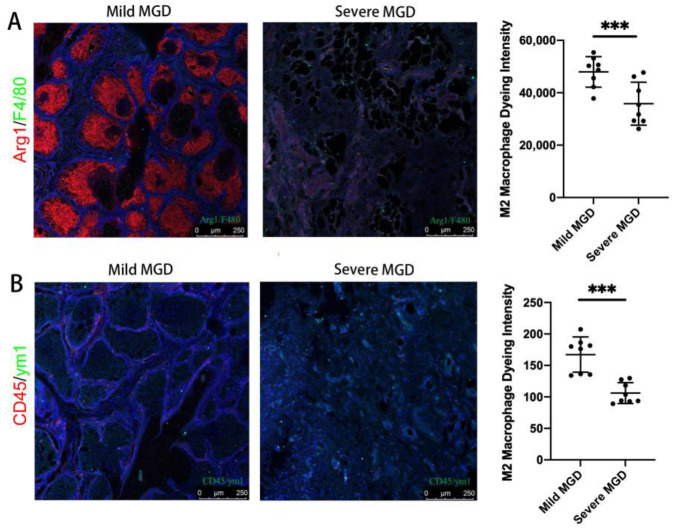
Immunofluorescence staining of M1 and M2 macrophages in MG tissues of mild and severe MGD patients. (**A**) The results showed that the expression levels of Arg1 (marker of M2) in MG tissues were significantly lower in severe MGD group than those in mild MGD group (*p* = 0.0041). (**B**) The results showed that the expression levels of ym1 (marker of M2) in MG tissues were significantly lower in the severe MGD group than those in the mild MGD group (*p* = 0.0001) (*** *p* < 0.001).

Oil red staining showed that ApoE^-/-^ MGD mice had a more obvious congestion of eyelid fat and a greater infiltration of inflammatory cells than normal mice, and most of them were M1 cells (Figure 2 and Figure 3A). The results of immunofluorescence staining showed that the expression levels of CD45, F4/80 and CD 86 (markers of M1 type macrophage) were significantly increased in ApoE^-/-^ mice (MGD mice) than those in WT mice (*p* < 0.0001, *p* < 0.0001, *p* = 0.0010). (Figure 3A–C). In MGD patients, acinar and glandular ducts were observed in the mild group, and acinar atrophy occurred in the severe group. Moreover, the results of immunofluorescence staining showed that the expression levels of Arg1 and ym1 (markers of M2) in MG tissues were significantly lower in severe MGD patients than those in mild MGD patients (*p* = 0.0041, *p* = 0.0001), which is consistent with the results of the macrophage infiltration in the MGD mouse model (Figure 4A,B). This indicated that the M1 macrophage involved the activation of the inflammatory response in MGD, whereas the M2 macrophage had the potential to inhibit the inflammatory response in MGD.

### 3.2. TIPE2 Expression Is Related to Macrophage Polarization

**Figure 5:**
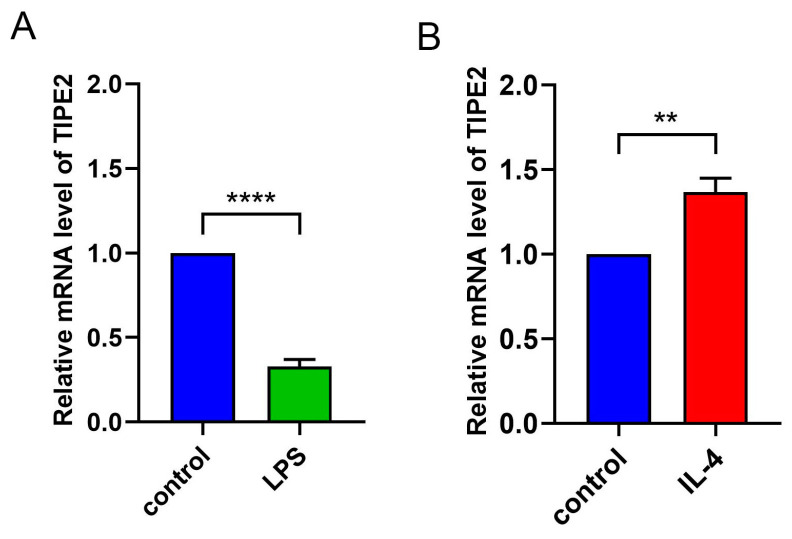
mRNA levels of TIPE2 in M1 and M2 macrophages. (**A**,**B**) The expression of TIPE2 mRNA was significantly decreased in M1 polarization induced by LPS (*p* < 0.0001), but increased in the process of polarization to the M2 type (*p* = 0.0016) (** *p* < 0.01, **** *p* < 0.0001).

We used classical (LPS) and alternative (IL-4) activated methods to simulate different macrophage polarization. The expression level of TIPE2 was detected in different activated macrophages. The results of the qRP-PCR showed that the expression level of TIPE2 decreased during M1-type polarization induced by LPS simulation (*p* < 0.0001), but increased during M2-type polarization induced by IL-4 simulation (*p* = 0.0016) (Figure 5). This indicated that TIPE2 was related to the polarization of macrophages.

### 3.3. TIPE2 Promotes the Polarization of M2 Macrophages

**Figure 6:**
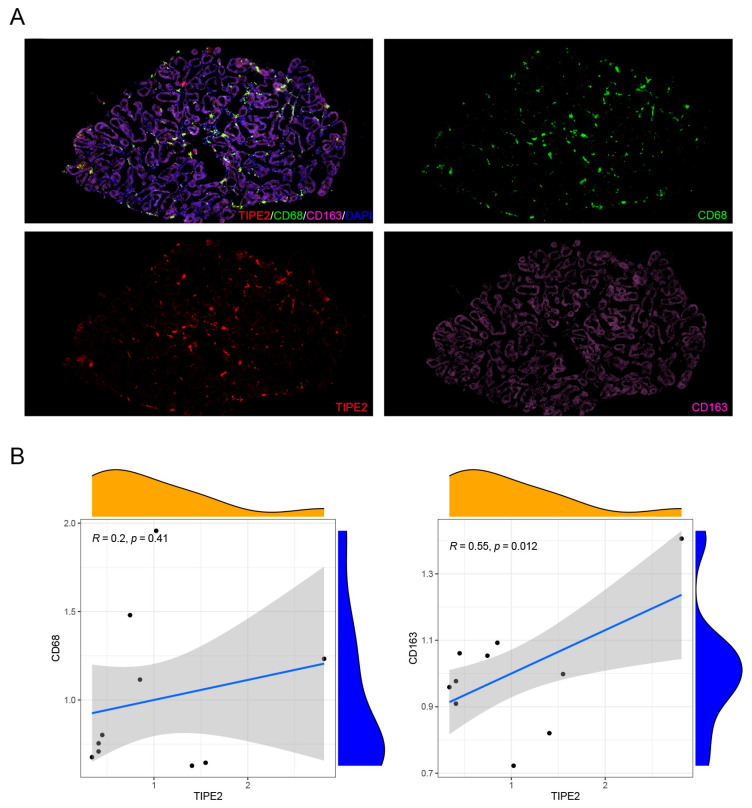
Immunofluorescence co-staining and the expression correlation between TIPE2 and markers of M1/M2 macrophages in MG tissues of MGD patients. (**A**,**B**) The results showed that the expression levels of CD68 were higher than CD163 in MG tissues of MGD. The expression level of TIPE2 was positively correlated with the expression level of CD163 (R = 0.55, *p* = 0.012). No significant correlation was found in the expression between TIPE2 and CD68 (R = 0.2, *p* = 0.41).

To verify the relationship between TIPE2 and the M2 polarization of macrophages, we detected the expression levels of TIPE2 and macrophage markers in MG tissues of MGD donors. The results of the immunofluorescence staining showed that TIPE2 was distributed around acinar cells in the MG tissue sections of MGD patients. The number of M1 macrophages (CD68) infiltrated in the MG tissue of MGD patients was much greater than that for the M2 type (CD163). Moreover, a positive correlation was found between the expression level of TIPE2 and CD163 (R = 0.550, *p* = 0.012), which indicated a potential role of TIPE2 in promoting the M2 polarization of macrophages in MG tissues (Figure 6A,B).

## 4. Discussion

In this research, we created an MGD mouse model and confirmed the inflammatory impact of M1 macrophages on MGD, the anti-inflammatory impact of M2 macrophages on MGD, and the fact that TIPE2 has a tendency towards the M2-directed polarization of MG macrophages.

Studies have shown that inflammatory factors such as IL-6, matrix metalloproteinase 9, interferon-γ and TNF-α are involved in the pathological process of MGD [15]. The secretion of inflammatory factors may activate the nuclear factor kappa-B (NF-κB) signaling pathway and the mitogen-activated protein kinase signaling pathway. These pathological mechanisms have been confirmed in studies such as those investigating the link between MGD and high-fat diets [16]. In the study of cardiovascular plaque stability in mice fed with a high-fat diet, it was found that TIPE2 can reduce macrophage apoptosis by reducing toll-like receptor 4 and the myeloid differentiation primary response gene (88), thus promoting plaque stability [17]. The observations of the ApoE^-/-^ MGD mouse model portray that a large quantity of inflammatory cells infiltrated around the MG acinar, and these inflammatory cells were mainly M1 macrophages. At the same time, we carried out tissue section staining of human meibomian glands, and confirmed that there was also macrophage infiltration in the meibomian gland of MGD patients. The severe group was dominated by M1 macrophages, and the mild group was dominated by M2 macrophages. Macrophages belong to leukocytes, which are phagocytic cells specialized by the innate immune system. Together with neutrophils, they become the first responders to infection, participating in the recognition, phagocytosis and degradation of cell debris and pathogens, as well as maintaining tissue homeostasis and tissue repair and remodeling [18]. Polarization is a simplified description of macrophage heterogeneity and plasticity, and is a continuous state involving multiple factors [19]. Macrophage polarization can be divided into classical activated M1 macrophages with pro-inflammatory effects and selective activated M2 macrophages with anti-inflammatory effects according to function and cell phenotype [20]. Polarization reversibility has key therapeutic value in diseases caused by M1/M2 imbalance. The signaling pathways for macrophage polarization to M1 include LPS/toll-like receptor 4 and the NF-κB/phosphatidylinositol 3 kinase pathways. M1 macrophages generally secrete pro-inflammatory factors through interferon-γ and LPS activation [21]. The signaling pathways of macrophage polarization to M2 include signal transducer and activator of transcription 6 and peroxisome proliferator-activated receptor γ. M2 macrophages can be activated by T helper 2 cytokines to secrete anti-inflammatory factors, which play a role in inhibiting inflammatory response and tissue repair [22]. Signaling pathway peroxisome proliferator-activated receptor γ specifically guides the lipogenesis of meibomian gland epithelial cells and inhibits the development of MGD [23]. M1 macrophages, characterized by CD68, CD86, and major compatibility complex II phenotype, play a pro-inflammatory role through the high expression of IL-12, -6, -1β, and other cytokines. M2 macrophages are characterized by CD68, CD209 and Ym1/2, and highly express cytokines and inflammatory medium such as IL-10 and transforming growth factor-β, which play anti-inflammatory and tissue repair roles [24]. Macrophages are dynamically converted under different microenvironment signals, thereby regulating the immune inflammatory response and affecting the outcome of disease. We observed different types of macrophages in the MG acinar of humans and MGD mice, and M1 type accounted for the majority, which was consistent with the pro-inflammatory effect of the M1 type mentioned above.

The study of the correlation between TIPE2 and immune function began in myeloid cells. Many studies have confirmed that TIPE2 is related to inflammation, and has the function of negatively regulating immune response and prompting apoptosis, which acts as an essential characteristic in maintaining immune homeostasis [25,26]. TIPE2 can negatively regulate the mammalian target of rapamycin complex 1 to regulate macrophage polarization by interfering with the excitation of NF-κB cell receptor-associated protease mammalian target of rapamycin complex 1, thereby participating in the inflammatory response [14]. Wynn et al. reported that TIPE2 promoted the differentiation of macrophages into the M2 type and was necessary for the immunosuppressive function of CD4+ and CD25+ Treg [27]. In the mice scald model, it was found that in the early stage of inflammatory response, the up-regulation of TIPE2 expression may lessen the intensity of the inflammatory response and avoid the damage caused by an excessive inflammatory response. In the TIPE2^-/-^ mice stroke model, it was discovered that TIPE2 deficiency increased the amount of neutrophils and macrophages, and elevated the scope of cerebral ischemic injury [28]. TIPE2 can treat atherosclerosis by inhibiting the NF-κB signaling pathway to decrease the inflammatory response of macrophages [29]. The over-expression of TIPE2 significantly lowered the proliferation of macrophages and inhibited the secretion of pro-inflammatory factors, becoming a new anti-inflammatory therapy to alleviate muscle weakness in patients with muscular dystrophy [30]. TIPE2 regulates macrophage M2 polarization and the low expression of pro-inflammatory factors by activating signal transducer and activator of transcription 3 and NF-κB signaling pathways to treat rheumatoid arthritis [26]. In this study, LPS was used to induce mouse bone-marrow-derived macrophages to M1 macrophages, and the expression level of TIPE2 was observed to decrease. After the induction of M2 macrophages with IL-4, the expression level of TIPE2 was observed to increase, indicating that TIPE2 can regulate macrophage polarization and effectively control the inflammatory response of MGD.

The incidence of MGD has increased year after year because of the ill habits surrounding eye care and diet, as well as air pollution and many other complex factors. MGD is characterized by its chronic and incurable nature, which has a serious impact on quality of life and creates socioeconomic pressures. Therefore, it is necessary to find new treatment strategies for MGD. This study observed the distribution of macrophages in the MG acinar of human and mouse MGD, as well as the characteristics of M2 macrophages in MGD regulated by TIPE2. However, there are still some limitations to this experiment. We have failed to demonstrate the complete mechanism by which TIPE2 regulates M2 macrophages in MGD, but based on all the data we have collected we believe it can work. Thus, there is room for the further study of this mechanism. In future studies, we will further explore the specific mechanism of TIPE2 in macrophage polarization.

## 5. Conclusions

This study proved that the M1 macrophage was the dominant group that infiltrated the MG tissue of MGD, and TIPE2 is a potential anti-inflammatory target for preventing the development of MGD by promoting M2-type polarization of macrophages. This indicates that TIPE2 might prevent the continuous progression of MGD, which has important clinical and social significance for MGD therapeutic strategies.

## Data Availability

The data used/or analyzed during the current study are available from the corresponding author upon reasonable request.
